# Severity Classification of Functional Impairment Based on ICF Qualifiers: A New Proposal for Assessing Individuals With Pulmonary Hypertension

**DOI:** 10.1002/pri.70090

**Published:** 2025-07-23

**Authors:** Jonathan Dalavina, Ivan Peres Costa, Etiene FarahTeixeira de Carvalho, Jonathan Luiz Silva, Soraia Micaela Silva, Luciana Maria Malosá Sampaio

**Affiliations:** ^1^ Postgraduate Program in Rehabilitation Sciences Nove de Julho University (UNINOVE) São Paulo Brazil

**Keywords:** functional capacity, international classification of functioning, pulmonary hypertension, six‐minute walk test, WHO/NYHA classification

## Abstract

**Background:**

Pulmonary Hypertension (PH) is a chronic condition that leads to progressive functional limitations, making the assessment of functional capacity essential for clinical management. This study aimed to classify PH patients based on the International Classification of Functioning, Disability, and Health (ICF) qualifiers using the Six‐Minute Walk Test (6MWT) and compare this classification with the World Health Organization Functional Classification (WHO‐FC).

**Methods:**

This observational study included 33 individuals with PH. Demographic data, pulmonary function, and 6MWT results were collected. Participants were classified according to ICF qualifiers (ranging from no impairment to complete impairment) and WHO‐FC. The association between classifications was tested using Fisher's exact test, considering the conceptual differences between them.

**Results:**

Most participants presented moderate functional impairment, with an average 6MWT distance of 431.5 ± 110 m, equivalent to 68% of the predicted value. Based on ICF qualifiers, 39% of patients had mild impairment, 42% moderate, and 18% severe impairment. However, no significant association was found between ICF qualifiers and WHO‐FC, reflecting the distinct conceptual frameworks of these classifications. Unlike WHO‐FC, which applies fixed cutoffs, the ICF‐based classification provides a more individualized assessment by incorporating the contrast between expected and actual performance in the 6MWT.

**Discussion:**

The use of ICF qualifiers enabled a more specific evaluation of functional capacity in PH patients, complementing rather than replacing WHO‐FC. This approach allows for a more individualized assessment, supporting targeted rehabilitation strategies and improving clinical decision‐making in PH management.

## 1 Introduction

Pulmonary Hypertension (PH) is a severe hemodynamic condition characterized by an elevation in mean pulmonary artery pressure (mPAP) above 20 mmHg (Kularatne et al. 2024). This condition leads to ventricular overload, reduced cardiac output, and decreased systemic oxygenation, manifesting primarily as progressive dyspnea, significant functional limitations, and in severe cases, heart failure that can be fatal (Simonneau et al. 2019; Galie et al. 2015). It is estimated that PH affects approximately 1% of the global population, with an incidence of 2–5 new cases per million adults annually (Prins and Thenappan 2016).

Therapeutic approaches for PH have shown improvements in patients' quality of life and exercise capacity (Miyamoto et al. 2000; Humbert et al. 2022). Assessments of functional capacity are crucial in this context, with exercise tests, such as cardiopulmonary exercise testing, Incremental Shuttle Walk Test (ISWT), and Incremental Step Test, being widely used. However, these tests have limitations due to the risk of cardiovascular complications and high costs (Kylhammar et al. 2018). The Six‐Minute Walk Test (6MWT) emerges as an effective alternative, being simple, low‐cost, and providing an objective measure of physical endurance, becoming the gold standard due to its high reliability, validity proven by correlations with VO_2_ max, and ability to detect clinically significant changes (ATS Committee on Proficiency Standards for Clinical Pulmonary Function Laboratories 2002). This test measures the distance a patient can walk in 6 minutes, reflecting their exercise tolerance. The results are interpreted based on the predicted values, which are calculated considering age, gender, height, and weight, enabling a standardized comparison of functional impairment (Brooks et al. 2003). Studies indicate that distances over 440 m in the 6MWT are associated with a lower risk of mortality (Simonneau et al. 2019; Souza et al. 2018).

The World Health Organization Functional Classification (WHO‐FC) for Pulmonary Hypertension is a fundamental tool in clinical practice and research, derived from the New York Heart Association (NYHA) system for pulmonary hypertension (McLaughlin et al. 2009). This classification is widely applied in disease management, guiding treatment decisions, monitoring disease progression, and supporting risk stratification—key factors in therapeutic strategies, particularly in pulmonary rehabilitation and pharmacological interventions. Among the criteria employed in this classification, performance on the 6MWT is a primary determinant, alongside other clinical parameters. However, the 6MWT‐based classification in the WHO‐FC applies standardized thresholds to all individuals with pulmonary hypertension, irrespective of their specific characteristics, potentially limiting the accuracy of individual functioning assessments.

Although extensively utilized, this approach does not account for interindividual variability in functional capacity. To address this limitation, we propose a novel classification based on the qualifiers of the International Classification of Functioning, Disability and Health (ICF), enabling a more precise and individualized assessment. Our approach contrasts the predicted 6MWT performance with the actual performance achieved, facilitating the quantification of functional impairment (Silva et al. 2024). This comparison allows for direct linkage to the ICF qualifiers, yielding a classification that more accurately reflects the patient's real‐world functioning. By overcoming the constraints of fixed WHO‐FC thresholds, this framework enhances sensitivity to individual patient characteristics, ultimately contributing to more precise diagnostics and optimized therapeutic decision‐making.

Thus, this study aims to propose a new classification of functional capacity in patients with pulmonary hypertension using the qualifiers of the ICF and comparing it with the WHO‐FC. This classification is based on the assessment of functional capacity through the 6MWT, in which the expected and actual performance are contrasted to quantify impairment and link it to the ICF qualifiers. Additionally, the study analyzes the association between these classifications to explore their relationship and potential implications for a more individualized evaluation of functioning. Given that the WHO‐FC applies standardized parameters while the ICF‐based approach provides a more individualized and realistic assessment of functioning, the association analysis aims to explore the relationship between these approaches, acknowledging that the lack of statistical association does not indicate a limitation but rather reflects the fundamental differences in the methodologies and parameters used.

## 2 Methods

### 2.1 Study Design and Participants

This is an observational study with a consecutive convenience sample composed of individuals with pulmonary arterial hypertension (PAH), referred to the Cardiopulmonary Rehabilitation Laboratory at UNINOVE in São Paulo, SP, Brazil.

The study was reviewed and approved by the Ethics Committee for Research with Human Beings at Universidade Nove de Julho (CoEP‐UNINOVE), São Paulo, Brasil (sob protocolo n°466/12 e Res. CNS 510/2016). All participants provided informed consent at the start of the study and were informed of the possibility of withdrawal from the research at any stage without any penalty. Adult individuals of both sexes, aged 20–70 years, with a confirmed diagnosis of PH, who had been under outpatient medical treatment for at least six months and had stable clinical conditions for three months (i.e., without exacerbations and on optimized medical therapy) were recruited. Prevalent cases were considered for the study, regardless of the time since diagnosis or the start date of care at the study site. Participants were excluded if they had significant musculoskeletal disease, claudication pain in the limbs, neurological or cognitive impairment, psychological or mood disorders that could affect their ability to perform clinical field tests.

The participants were grouped based on levels of functional impairment according to the generic ICF qualifiers, ranging from 0.0 to 0.4. These qualifiers reflect the degree of impairment and are categorized as: no impairment, mild, moderate, severe, and complete impairment (World Health Organization 2001). Groups were formed according to the percentage of the predicted values in the Six‐Minute Walk Test (6MWT) (Brooks et al. 2003). Facilitating comparisons across functioning levels and enabling a more precise analysis of patients' functional capacity (Table 1).

**TABLE 1:** Parallel between ICF qualifiers and 6MWT obtained results.

ICF qualifiers	Qualitative description of ICF qualifiers	% Of predicted 6MWT (*n* = 33)
0.0	No problem (0%–4%)	96%–100% predicted (*n* = 1)
0.1	Mild problem (5%–24%)	96%–100% predicted (*n* = 12)
0.2	Moderate problem (25%–49%)	51%–75% predicted (*n* = 14)
0.3	Severe problem (50%–95%)	5%–50% predicted (*n* = 6)
0.4	Complete problem (96%–100%)	0%–4% predicted (*n* = 0)

Abbreviations: % predicted: Percentage of the predicted distance in the Six‐Minute Walk Test; 6MWT: six‐minute walk test; ICF: International Classification of Functioning, Disability and Health.

### 2.2 Assessment Methods

Body mass index (BMI) was determined using an anthropometric scale and a stadiometer (Welmy) with the participant barefoot. Body weight (kg) and height (m) were measured, and BMI was calculated as kg/m^2^.

Pulmonary function testing followed the 2002 Spirometry Guidelines and included the measurement of forced vital capacity (FVC) and Forced Expiratory Volume in the first second (FEV1), using reference values for the Brazilian population (Pereira et al. 1992). The following variables were recorded for analysis: forced vital capacity (FVC), forced expiratory volume in the first second (FEV1), peak expiratory flow (PEF), forced expiratory flow 25%–75% (FEF25%‐75%), and the FEV1/FVC ratio. Values were expressed as absolute values and percentages of the predicted value (%pred) for the Brazilian population.

The Six‐Minute Walk Test (6MWT) aims to measure the maximum distance a patient can walk on a flat, straight surface within 6 minutes, quantifying functional mobility in meters. The 6MWT was conducted according to the guidelines established by the American Thoracic Society (ATS) (Brooks et al. 2003) in a 30‐m corridor marked by cones. Volunteers were instructed to walk at the highest tolerable speed for 6 minutes. The assessor provided standardized verbal encouragement every minute: “You are doing well, there are x (1, 2, 3, 4, or 5) minutes left.”

Following ATS recommendations, participants completed the test twice on the same day, with at least 30 min of rest or enough time to restore baseline vital signs between trials. At the start and end of each test, systolic (SBP) and diastolic blood pressure (DBP), heart rate (HR) in beats per minute (bpm), peripheral oxygen saturation (SpO_2_), and perceived exertion for lower limbs (BORG MMII) and dyspnea (BORG D) were assessed using the modified Borg scale, with higher scores indicating greater effort (Brooks et al. 2003). Heart rate and SpO_2_ were monitored throughout the test using a pulse oximeter with a 512 Respironics sensor (Philips, Massachusetts, USA) along with perceived exertion (BORG MMII and BORG D).

Blood pressure (BP) was measured according to Brazilian hypertension guidelines using a mercury manometer with a vertical scale from 0 to 300 mmHg (UNITEC) and a stethoscope (Littmann). The test was interrupted in cases of discomfort, presyncope, nausea, significant dyspnea, extreme fatigue, chest pain, HR above 85% of the predicted maximum, or SpO2 ≤ 80% (Brooks et al. 2003; Dourado 2011).

The equations proposed by Dourado (2011) [DPmTC6pred = 299.296—(2.728 × age)—(2.160 × weight) + (361.731 × height) + (56.386 × sex), where male = 1, female = 0] were used to compare the distance covered by volunteers with predicted values for the Brazilian population (Dourado 2011).

### 2.3 WHO Functional Classification for Pulmonary Hypertension

The World Health Organization Functional Classification (WHO‐FC), based on the New York Heart Association (NYHA) system for PH, is a fundamental tool in clinical practice and research, with broad applications in disease management (Table 2) (McLaughlin et al. 2009). It guides treatment, monitors progression, and defines risk stratification, crucial for therapeutic decisions, particularly in Pulmonary Rehabilitation and medication selection (Humbert et al. 2022; Rohde et al. 2023). In cases of advanced PH, this classification guides the use of more aggressive therapies, essential for improving patient prognosis and quality of life.

**TABLE 2:** World health organization functional assessment and New York heart association (WHO/NYHA) [McLaughlin et al. 2009; Rubin and American College of Chest Physicians 2004].

Functional class	Description
Class I	Patients with PH but without resulting limitation of physical activity. Ordinary physical activity does not cause undue dyspnea or fatigue, chest pain, or near syncope.
Class II	Patients with PH resulting in slight limitation of physical activity. They are comfortable at rest. Ordinary physical activity causes undue dyspnea or fatigue, chest pain, or near syncope.
Class III	Patients with PH resulting in marked limitation of physical activity. They are comfortable at rest. Less than ordinary activity causes undue dyspnea or fatigue, chest pain, or near syncope.
Class IV	Patients with PH with inability to carry out any physical activity without symptoms. These patients manifest signs of right‐heart failure. Dyspnea and/or fatigue may even be present at rest. Discomfort is increased by any physical activity.

Abbreviation: PH: pulmonary hypertension.

Additionally, this classification of WHO‐FC is widely used in clinical research for patient stratification, providing a reliable assessment of PH severity and prognosis. Inspired by the NYHA system for heart failure, the WHO/NYHA patients were divided into four classes based on symptoms and physical activity limitations, providing a detailed view of functional impairment (Rubin and American College of Chest Physicians 2004). This approach enables more personalized management with treatment adjusted according to disease severity, enhancing symptom control and improving quality of life for PH patients, promoting more targeted and effective clinical intervention.

## 3 International Classification of Functioning, Disability, and Health (ICF)

The ICF, developed by the WHO in 2001, adopts a biopsychosocial approach that expands health assessment by considering, in addition to clinical conditions, the environmental and social factors that impact individuals' lives (Cieza et al. 2002). This framework provides an integrated view of health, enabling a more comprehensive understanding of individuals' limitations and capabilities in various contexts (Monteiro et al. 2021; Organização Mundial da Saúde Organização Panamericana da Saúde 2003).

It is divided into two main parts. The first part addresses functioning and disability, subdivided into body functions and structures, and activities and participation; the second part deals with contextual factors, which include both environmental and personal factors that directly influence functioning. This dual structure allows for a holistic and detailed assessment of health and the impact of health conditions on the individual's daily life (Monteiro et al. 2021).

To describe health status and functioning, the ICF uses alphanumeric codes, with letters (b, s, d, e) representing the main components: body functions (b), body structures (s), activities and participation (d), and environmental factors (e). These codes are accompanied by qualifiers indicating the severity of the issue on a scale of intensity, enabling precise and personalized analysis (Cieza et al. 2002). The ICF complements the International Classification of Diseases (ICD‐10), broadening the scope of assessment to include functional and social aspects not covered by clinical diagnosis. The WHO emphasizes that the ICF and ICD‐10 complement each other: while the ICD‐10 provides the diagnosis, the ICF describes functioning and disability, offering an integrated and personalized view of health (Organização Mundial da Saúde Organização Panamericana da Saúde 2003).

The application of the ICF standardizes the language used by health professionals globally, allowing comparisons across populations and facilitating more effective interventions^19^. This tool has been essential in evaluating and monitoring chronic diseases, allowing the use of various instruments in diverse health contexts (Singh et al. 1992).

### 3.1 Functional Classification Based on ICF Qualifiers for 6MWT Assessment

To categorize the results obtained in the 6MWT, we used the generic ICF qualifiers, which range from 0.0 to 0.4, with a higher qualifier indicating greater problem severity. Thus, a qualifier of 0.0 indicates no problem, while a qualifier of 0.4 indicates a complete problem. There are also options to qualify ICF codes with 0.8 (unspecified) or 0.9 (not applicable). The impairment can also be qualitatively analyzed by observing the percentage of individual impairment (Table 1) (World Health Organization 2002).

To establish the relationship between the 6MWT results and ICF qualifiers, we obtained the values of the distance covered and analyzed the distance the individual walked relative to the predicted distance for their age, weight, height, and gender. Given the inverse relationship between the ICF qualifiers (the higher the qualifier, the greater the impairment) and the 6MWT results (the greater the distance covered, the lesser the impairment), we estimated the relationship between the 6MWT results and the ICF qualifiers to facilitate the practical application of this proposal in clinical settings (Peres Costa et al. 2022; Dourado; Dos Santos et al. 2022). Two displays this relationship, showing the generic scale of ICF qualifiers alongside the corresponding results from the 6MWT.

### 3.2 Statistical Analysis

The characterization of the sample and the distribution of the obtained scores were summarized according to the result of data distribution normality assessed using the Shapiro‐Wilk test. Parametric measures were expressed through central tendency (mean) and dispersion (standard deviation), and categorical variables were presented in frequency. Non‐parametric variables were summarized using median and interquartile ranges.

Volunteers were matched by age and BMI. For comparative analysis, severity groups were formed based on the results obtained in the 6MWT (mild/no impairment vs. moderate vs. severe), comparing the distance walked by each group. For this, a One‐Way ANOVA test was used to compare the groups with Bonferroni post hoc analysis applied.

To assess the correlation between the quantitative ICF qualifiers and the WHO functional classes, data were organized in a contingency table, which displays the observed and expected frequencies for each ICF qualifier and functional class. Participants were grouped as shown in Table 3. To verify the association between these variables, Pearson's Chi‐Square test was applied. However, due to low expected counts (less than 5 in 87.5% of the cells), Fisher's Exact Test was required, particularly suitable for low‐frequency situations. This dual approach allowed for a more reliable analysis of the association between variables. The statistical package used was SPSS version 22, and for analysis, *p* = 0.05 was considered significant.

**TABLE 3:** Categorization according to the qualifiers of ICF and WHO‐FC.

Category	Participants
ICF qualifiers	
0.0 (no problem)	1
0.1 (mild problem)	12
0.2 (moderate problem)	13
0.3 (severe problem)	7
WHO‐FC	
Class I	2
Class II	6
Class III	22
Class IV	3

Abbreviations: ICF: International Classification of Functioning, Disability and Health; WHO‐FC: World Health Organization Functional Classification.

## 4 Results

The sample consisted of 33 individuals, with thirteen (39.39%) presenting mild/no impairment, fourteen (42.42%) moderate impairment, and six (18.18%) classified as having severe impairment. No participant was classified as having complete impairment in functional capacity. Table 4 provides more detailed demographic and clinical characteristics of the studied population, summarizing the comparison among the three groups formed according to the generic ICF qualifiers classification (mild/no impairment, moderate, and severe). The individuals studied were considered homogeneous regarding anthropometric data and clinical characteristics.

**TABLE 4:** Demographic and clinical characteristics of participants.

Variable	All participants (*n* = 33)	Severe (*n* = 6)	Moderate (*n* = 14)	Mild/no problem (*n* = 13)
Sex F/M	24/9	5/1	10/4	10/3
Age, y	39,39 ± 17,29	35,16 ± 23,77	33,85 ± 13,64	47,30 ±16,52
Weight, kg	67,87 ± 15,74	62,96 ± 24,46	71,42 ± 15,26	66,31 ± 12,26
Height, m	1,59 ± 0,08	1,55 ± 0,11	1,63 ± 0,04	1,58 ± 0,08
BMI, kg/m2	26,43 ± 5,72	25,51 ± 7,78	26,73 ± 5,77	26,55 ± 5,30
WHO‐FC				
I *n*, (%)	2 (6,06%)	0 (0%)	2 (14,28%)	0
II *n*, (%)	6 (18,18%)	0 (0%)	3 (21,42%)	3 (21,42%)
III *n*, (%)	22 (66,66%)	4 (66,66%)	8 (57,14%)	10 (78,58%)
IV *n*, (%)	3 (9,09%)	2 (33,33%)	1 (7,14%)	0 (%)
PAPs (mmHg)	69,63 ± 16,97	73,33 ± 12,92	66,92 ± 19,49	70,84 ± 17,16
Pulmonary function				
FVC % pred	77,97 ± 24,73	75,77 ± 30,28	77,84 ± 16,58	77,76 ± 31,62
FEV1% pred	71,97 ± 20,37	74,06 ± 25,14	74,79 ± 17,91	67,97 ± 22,40
6MWD (m)	431,54 ± 110,19	253,16 ± 50,80	426,25 ± 72,12	519,57 ± 48,44
6MWT ‐predicted distance (m)	637,44 ± 59,34	637,43 ± 53,25	660,46 ± 55,51	612,65 ± 62,30
% Predicted ‐ 6MWT (m)	68,02 ± 17,89	39,53 ± 5,51	64,35 ± 8,44	85,13 ± 7,19

Abbreviations: 6MWD: 6‐min walking distance; 6MWT: 6‐min walking test; BMI: Body Mass Index; FEV1: Forced Expired Volume in the first second; FVC: Forced Vital Capacity; PAPs: systolic pulmonary arterial pressure; WHO‐FC: World Health Organization Functional Classification.

Impairment on the distance walked (*F* = 48.584; *p* = 0.001) and on the percentage of predicted distance (*F* = 71.304; *p* = 0.001) showed statistically significant differences in the 6MWT distance of the mild/no impairment group compared with the other groups (*F* = 36.277; *p* < 0.001). Bonferroni post hoc analysis indicated that on average, the distance walked by the mild/no impairment group differed from that of the moderate and severe groups (*p* = 0.001; *p* = 0.001, respectively) as well as between the moderate and severe groups (*p* = 0.001).

Analysis of the correlation between ICF qualifiers and WHO/NYHA classes for PH showed no statistically significant association between the variables, as indicated by Pearson's chi‐square test (*χ* ^2^ = 9.777; df = 9; *p* = 0.369). Fisher's Exact Test confirmed this lack of significance (*p* = 0.388), reinforcing the independence between functional impairment levels (ICF) and WHO/NYHA in the studied sample. These results suggest that patients can exhibit different levels of functional impairment regardless of their classification on the WHO scale, indicating that these two measures seem unrelated in this context.

## 5 Discussion

Most of the sample presented with mild or moderate functional impairment, according to ICF qualifiers, with only 18.18% of participants classified as severely impaired and none as completely impaired. This distribution suggests that, despite a diagnosis of pulmonary hypertension (PH), most patients retain a certain degree of functionality.

Comparative analysis between groups revealed statistically significant differences in both the absolute 6MWT distance and the percentage of predicted distance, confirming the test's sensitivity in distinguishing between levels of functional impairment. Patients classified with mild or no impairment walked significantly longer distances—for instance, those with mild impairment (qualifier 0.1) reached 76%–95% of the predicted distance, while those with severe impairment (qualifier 0.3) covered only 5%–50%. These findings reinforce the effectiveness of the 6MWT as a tool for assessing functional limitations in PH.

Regarding clinical and anthropometric variables, patients with more severe impairment had lower average weight and BMI although the overall sample showed an overweight profile. These results suggest that in more advanced disease stages, body mass loss may occur due to malnutrition or systemic involvement. The mean pulmonary artery systolic pressure (PASP) was elevated (69.63 mmHg) but showed no significant correlation with the 6MWT distance, underscoring the limitations of isolated hemodynamic variables in representing functional capacity. No sex‐based differences were observed, indicating similar functional limitations in men and women with PH.

Despite the alignment between 6MWT results and ICF‐defined levels of functional impairment, no statistical association was found between ICF qualifiers and the WHO/NYHA Functional Classification. Variability across ICF qualifiers (Table 3) may reflect factors influencing individual functioning, even among patients similarly classified by WHO/NYHA. Unlike the WHO classification, which relies on fixed thresholds based on standardized test outcomes, the ICF‐based approach offers a more individualized assessment. It compares expected and actual performance, yielding a more precise and patient‐centered evaluation. For example, patients classified as WHO Class III may show mild (0.1) or severe (0.3) impairment under the ICF, depending on how their actual performance compares to expected values. This highlights the greater specificity of the ICF‐based model, which captures individual variations more effectively than the generalized WHO‐FC.

Patients who respond positively to treatment and improve their exercise capacity may be assigned lower ICF qualifiers, even if their WHO class remains unchanged. In contrast, those with disease progression or poor treatment response may exhibit higher ICF qualifiers, indicating greater impairment. This shows how the ICF framework more accurately reflects functional changes by focusing on the gap between expected and actual performance rather than relying on fixed parameters.

The combined use of the 6MWT and ICF provides a more integrated and nuanced analysis of PH's functional impact. This study found that patients in more advanced NYHA classes (III and IV) walked significantly shorter distances, indicating more severe limitations and increased mortality risk—emphasizing the value of the 6MWT in risk stratification and therapeutic planning. Furthermore, incorporating the ICF into pulmonary rehabilitation allows for more accurate identification of specific limitations, such as dyspnea and reduced aerobic capacity, enabling tailored interventions and contributing to clinical improvements in VO_2_ max and cardiac output (Spruit et al. 2013; Mainguy et al. 2010; Farber et al. 2015).

This study highlights the potential of an ICF‐based classification to provide a more individualized and precise assessment of functional capacity in patients with pulmonary hypertension. By comparing expected and actual performance on the 6MWT, this approach moves beyond the fixed parameters of the WHO/NYHA, offering a classification that better reflects each patient's real functioning. Although no direct association was found between the two classification systems, this result reinforces their conceptual differences: while the WHO‐FC applies standardized cutoffs, the ICF qualifiers capture functional impairment in a more specific and patient‐centered manner. These findings support the relevance of integrating an ICF‐based perspective into functional assessment, paving the way for future research to explore its applicability in clinical decision‐making and rehabilitation strategies.

This study introduces an ICF‐based classification for pulmonary hypertension that contrasts expected and actual 6MWT performance, providing a more individualized assessment of functional capacity. Unlike the WHO classification, which applies fixed parameters, this approach captures patient‐specific functioning, offering greater precision in functional evaluation. Although no association was found between the two classifications, this result underscores their distinct conceptual frameworks. The proposed method enhances functional assessment, supporting more targeted rehabilitation strategies and contributing to a more individualized approach in pulmonary hypertension management. It is important to note that the intention is not to replace the WHO classification but to complement it by offering a more personalized and dynamic evaluation of functional capacity.

### 5.1 Study Limitations

This study has certain limitations. The cross‐sectional design restricts the ability to infer causality or to monitor changes in functional capacity over time. Additionally, the relatively small sample size and the recruitment of participants from a single center may limit the generalizability of the findings. Furthermore, the lack of statistical association between ICF qualifiers and WHO‐FC likely reflects conceptual divergences between these instruments rather than methodological flaws. These limitations were mitigated through the application of strict inclusion criteria, standardized administration of the six‐minute walk test (6MWT) in accordance with international guidelines, and the use of reference equations specific to the Brazilian population. Moreover, the classification model proposed—grounded in ICF qualifiers—aims to complement existing approaches by providing a more individualized and context‐sensitive assessment of functional capacity in individuals with pulmonary hypertension.

### 5.2 Implications for Physiotherapy Practice

The ICF‐based classification provides a more specific evaluation of functional capacity by contrasting expected and actual 6MWT performance, allowing targeted rehabilitation strategies. A classification reflecting actual functioning enables precise monitoring of disease progression and treatment response, improving exercise prescription and pulmonary rehabilitation strategies. The use of ICF qualifiers enhances interdisciplinary communication, aligning physiotherapy interventions with broader clinical management for a comprehensive, patient‐centered approach.

## Author Contributions

J.D. and L.M.M.S. conceptualized the study, developed the research protocol, synthesized, and analyzed the data. I.P.C., E.F.T.C., J.L.S. data collection. J.D., L.M.M.S. and S.M.S. discussed the results and contributed to the final manuscript. J.D. wrote the manuscript. L.M.M.S. and S.M.S. helped on the manuscript final version. All authors reviewed the manuscript and agreed to its published version.

## Ethics Statement

(CoEP‐UNINOVE), São Paulo, Brazil (under protocol No. 466/12).

## Consent

(CoEP‐UNINOVE), São Paulo, Brazil (under protocol No. 466/12).

## Conflicts of Interest

The authors declare no conflicts of interest.

## Data Availability

The data that support the findings of this study are available from the corresponding author upon reasonable request.

## References

[pri70090-bib-0001] ATS Committee on Proficiency Standards for Clinical Pulmonary Function Laboratories . 2002. “ATS Statement: Guidelines for the Six‐Minute Walk Test.” American Journal of Respiratory and Critical Care Medicine 166, no. 1: 111–117: Erratum in: Am J Respir Crit Care Med. 2016;193(10):1185. 10.1164/rccm.19310erratum. 12091180

[pri70090-bib-0004] Brooks, D. , S. Solway , and W. J. Gibbons . 2003. “ATS Statement on Six‐Minute Walk Test.” American Journal of Respiratory and Critical Care Medicine 167, no. 9 May: 1287: PMID: 12714344. 10.1164/ajrccm.167.9.950. 12714344

[pri70090-bib-0006] Cieza, A. , T. Brockow , T. Ewert , et al. 2002. “Linking Health‐Status Measurements to the International Classification of Functioning, Disability and Health.” Journal of Rehabilitation Medicine 34, no. 5: 205–210. 10.1080/165019702760279189. 12392234

[pri70090-bib-0007] Dos Santos, H. M. , L. C. de Oliveira , S. R. Bonifácio , et al. 2022. “Use of the International Classification of Functioning, Disability and Health (ICF) to Expand and Standardize the Assessment of Quality‐Of‐Life Following a Stroke: Proposal for the Use of Codes and Qualifiers.” Disability & Rehabilitation 44, no. 24 Dec: 7449–7454: Epub 2021 Nov 9. PMID: 34752176. 10.1080/09638288.2021.1995055. 34752176

[pri70090-bib-0008] Dourado, V. Z . 2011. “Reference Equations for the 6‐Minute Walk Test in Healthy Individuals.” Arquivos Brasileiros de Cardiologia 96, no. 6: 128–138. 21359481

[pri70090-bib-0009] Farber, H. W. , D. P. Miller , M. D. McGoon , A. E. Frost , W. W. Benton , and R. L. Benza . 2015. “Predicting Outcomes in Pulmonary Arterial Hypertension Based on the 6‐Minute Walk Distance.” Journal of Heart and Lung Transplantation 34, no. 3 Mar: 362–368: Epub 2014 Aug 28. PMID: 25312386. 10.1016/j.healun.2014.08.020. 25312386

[pri70090-bib-0010] Galie, N. , M. Humbert , J. L. Vachiery , et al. 2015. “ESC/ERS Guidelines for the Diagnosis and Treatment of Pulmonary Hypertension.” European Heart Journal. 10.1093/eurheartj/ehv317. 26837729

[pri70090-bib-0012] Humbert, M. , G. Kovacs , M. M. Hoeper , et al. 2022. “ESC/ERS Guidelines for the Diagnosis and Treatment of Pulmonary Hypertension: Developed by the Task Force for the Diagnosis and Treatment of Pulmonary Hypertension of the European Society of Cardiology (ESC) and the European Respiratory Society (ERS).” European Heart Journal 43, no. 38: 3618–3731. 10.1093/eurheartj/ehac237. 36017548

[pri70090-bib-0014] Kularatne, M. , C. Gerges , M. Jevnikar , M. Humbert , and D. Montani . 2024. “Updated Clinical Classification and Hemodynamic Definitions of Pulmonary Hypertension and Its Clinical Implications.” Journal of Cardiovascular Development and Disease 11, no. 3: 78. 10.3390/jcdd11030078. 38535101 PMC10971453

[pri70090-bib-0015] Kylhammar, D. , B. Kjellström , C. Hjalmarsson , et al. 2018. “A Comprehensive Risk Stratification at Early Follow‐Up Determines Prognosis in Pulmonary Arterial Hypertension.” European Heart Journal 39, no. 47: 4175–4181. 10.1093/eurheartj/ehx257. 28575277

[pri70090-bib-0016] Mainguy, V. , F. Maltais , D. Saey , et al. 2010. “Effects of a Rehabilitation Program on Skeletal Muscle Function in Idiopathic Pulmonary Arterial Hypertension.” Journal of Cardiopulmonary Rehabilitation and Prevention 30, no. 5: 319–323. 10.1097/hcr.0b013e3181d6f962. 20410828

[pri70090-bib-0018] McLaughlin, V. V. , S. L. Archer , D. B. Badesch , et al. 2009. “ACCF/AHA 2009 Expert Consensus Document on Pulmonary Hypertension: A Report of the American College of Cardiology Foundation Task Force on Expert Consensus Documents and the American Heart Association Developed in Collaboration With the American College of Chest Physicians; American Thoracic Society Inc.; and the Pulmonary Hypertension Association.” Journal of the American College of Cardiology 53, no. 17: 1573–1619. 10.1016/j.jacc.2009.01.004. 19389575

[pri70090-bib-0019] Miyamoto, S. , N. Nagaya , T. Satoh , et al. 2000. “Clinical Correlates and Prognostic Significance of Six‐Minute Walk Test in Patients With Primary Pulmonary Hypertension: Comparison With Cardiopulmonary Exercise Testing.” American Journal of Respiratory and Critical Care Medicine 161, no. 2: 487–492. 10.1164/ajrccm.161.2.9906015. 10673190

[pri70090-bib-0020] Monteiro, I. O. , N. S. C. Oliveira , J. A. Ruaro , D. S. Dantas , and S. M. A. Câmara . 2021. “Convergent Validity and Reproducibility of the International Classification of Functioning, Disability and Health (ICF) Core Set for the Physical Health of Community‐Dwelling Older Adults.” Brazilian Journal of Physical Therapy 25, no. 5: 563–572. 10.1016/j.bjpt.2021.02.007. 33731278 PMC8536858

[pri70090-bib-0021] Organização Mundial da Saúde, Organização Panamericana da Saúde . 2003. Classificação Internacional de Funcionalidade, Incapacidade e Saúde. Edusp.

[pri70090-bib-0022] Pereira, C. , S. Barreto , and J. Simões . 1992. “Valores de referência para espirometria em uma amostra da população brasileira adulta.” Jornal de Pneumologia 18: 10–22.

[pri70090-bib-0023] Peres Costa, I. , S. M. Silva , G. Alves da Silva , et al. 2022. “Shuttle Walk Test Categorization According to the Qualifiers of the International Classification of Functioning, Disability, and Health: Proposal for Use in Patients With Difficult‐To‐Treat Asthma, a Cross‐Sectional Study.” Physiotherapy Theory and Practice 39, no. 9: 1888–1895. 10.1080/09593985.2022.2063212. 35414340

[pri70090-bib-0024] Prins, K. W. , and T. Thenappan . 2016. “World Health Organization Group I Pulmonary Hypertension: Epidemiology and Pathophysiology.” Cardiology Clinics 34, no. 3: 363–374. 10.1016/j.ccl.2016.04.001. 27443134 PMC4959804

[pri70090-bib-0025] Rohde, L. E. , A. Zimerman , M. Vaduganathan , et al. 2023. “Associations Between New York Heart Association Classification, Objective Measures, and Long‐Term Prognosis in Mild Heart Failure: A Secondary Analysis of the PARADIGM‐HF Trial.” JAMA Cardiology 8, no. 2: 150–158. 10.1001/jamacardio.2022.4427. 36477809 PMC9857149

[pri70090-bib-0026] Rubin, L. J. , and American College of Chest Physicians . 2004. “Diagnosis and Management of Pulmonary Arterial Hypertension: ACCP Evidence‐Based Clinical Practice Guidelines.”supplement Chest 126, no. S1 July: 7S–10S: PMID: 15249491. 10.1378/chest.126.1_suppl.7S. 15249491

[pri70090-bib-0027] Silva, C. A. V. S. , G. B. Pires , G. S. Pereira , S. M. Silva , and L. M. M. Sampaio . 2024. “Disability After Breast Cancer Surgery: Proposal of Linkage Between the International Classification of Functioning, Disability and Health and the Grocery Shelving Task Test.” Physiotherapy Research International 29, no. 1 Jan: e2057: Epub 2023 Oct 15. PMID: 37839015. 10.1002/pri.2057. 37839015

[pri70090-bib-0029] Simonneau, G. , D. Montani , D. S. Celermajer , et al. 2019. “Haemodynamic Definitions and Updated Clinical Classification of Pulmonary Hypertension.” European Respiratory Journal 53, no. 1: 1801913. 10.1183/13993003.01913-2018. 30545968 PMC6351336

[pri70090-bib-0030] Singh, S. J. , M. D. L. Morgan , S. Scott , D. Walters , and A. E. Hardman . 1992. “Development of a Shuttle Walking Test of Disability in Patients With Chronic Airways Obstruction.” Thorax 47, no. 12: 1019–1024. 10.1136/thx.47.12.1019. 1494764 PMC1021093

[pri70090-bib-0031] Souza, R. , R. N. Channick , M. Delcroix , et al. 2018. “Association Between Six‐Minute Walk Distance and Long‐Term Outcomes in Patients With Pulmonary Arterial Hypertension: Data From the SERAPHIN Randomized Trial.” PLoS One 13, no. 3: e0193226. 10.1371/journal.pone.0193226. 29590122 PMC5873992

[pri70090-bib-0032] Spruit, M. A. , S. J. Singh , C. Garvey , et al. 2013. “An Official American Thoracic Society/European Respiratory Society Statement: Key Concepts and Advances in Pulmonary Rehabilitation.” American Journal of Respiratory and Critical Care Medicine 188, no. 8: e13–e64: Erratum in: Am J Respir Crit Care Med. 2014;189(12):1570. 10.1164/rccm.201309-1634ST. 24127811

[pri70090-bib-0033] World Health Organization . 2001. International Classification of Functioning, Disability and Health: ICF. World Health Organization.

[pri70090-bib-0035] World Health Organization . 2002. Towards a Common Language for Functioning, Disability and Health – ICF (WHO/EIP/GPE/CAS/01.3). World Health Organization.

